# Evidence of a Redox-Dependent Regulation of Immune Responses to Exercise-Induced Inflammation

**DOI:** 10.1155/2016/2840643

**Published:** 2016-11-15

**Authors:** Alexandra Sakelliou, Ioannis G. Fatouros, Ioannis Athanailidis, Dimitrios Tsoukas, Athanasios Chatzinikolaou, Dimitris Draganidis, Athanasios Z. Jamurtas, Christina Liacos, Ioannis Papassotiriou, Dimitrios Mandalidis, Kimon Stamatelopoulos, Meletios A. Dimopoulos, Asimina Mitrakou

**Affiliations:** ^1^Department of Clinical Therapeutics, Medical School, University of Athens, 11527 Athens, Greece; ^2^School of Physical Education and Sport Sciences, University of Thessaly, Karies, 42100 Trikala, Greece; ^3^School of Physical Education and Sport Sciences, Democritus University of Thrace, 69100 Komotini, Greece; ^4^Department of Toxicology, Medical School, University of Athens, 11527 Athens, Greece; ^5^Department of Clinical Biochemistry, “Aghia Sophia” Children's Hospital, 11527 Athens, Greece; ^6^School of Physical Education and Sport Science, University of Athens, Athens, Greece

## Abstract

We used thiol-based antioxidant supplementation (n-acetylcysteine, NAC) to determine whether immune mobilisation following skeletal muscle microtrauma induced by exercise is redox-sensitive in healthy humans. According to a two-trial, double-blind, crossover, repeated measures design, 10 young men received either placebo or NAC (20 mg/kg/day) immediately after a muscle-damaging exercise protocol (300 eccentric contractions) and for eight consecutive days. Blood sampling and performance assessments were performed before exercise, after exercise, and daily throughout recovery. NAC reduced the decline of reduced glutathione in erythrocytes and the increase of plasma protein carbonyls, serum TAC and erythrocyte oxidized glutathione, and TBARS and catalase activity during recovery thereby altering postexercise redox status. The rise of muscle damage and inflammatory markers (muscle strength, creatine kinase activity, CRP, proinflammatory cytokines, and adhesion molecules) was less pronounced in NAC during the first phase of recovery. The rise of leukocyte and neutrophil count was decreased by NAC after exercise. Results on immune cell subpopulations obtained by flow cytometry indicated that NAC ingestion reduced the exercise-induced rise of total macrophages, HLA^+^ macrophages, and 11B^+^ macrophages and abolished the exercise-induced upregulation of B lymphocytes. Natural killer cells declined only in PLA immediately after exercise. These results indicate that thiol-based antioxidant supplementation blunts immune cell mobilisation in response to exercise-induced inflammation suggesting that leukocyte mobilization may be under redox-dependent regulation.

## 1. Introduction

Skeletal muscle trauma may be induced by diseases (e.g., cancer, dystrophies), sepsis, toxin injection, mechanical destruction, freezing and/or intense lengthening stretches causing tissue breakdown, subcellular injury, extensive proteolysis, and muscle wasting that result in deterioration of skeletal muscle function [1]. Exercise-induced muscle microtrauma instigates an inflammatory reaction which is followed by a healing and/or regeneration phase that is associated with activation of myogenic cells, also known as satellite cells, and augmented protein synthesis that restores muscle's physiological structure and function [2]. The inflammatory and repair phases are interconnected; that is, they develop in succession while suppression of the former may hinder muscle's recovery [3]. Powerful eccentric exercise generates repetitive lengthening stretches of skeletal muscle that results in considerable disruption of its myofiber that is followed by an inflammatory response characterized by cytokine discharge that trigger an accumulation and/or infiltration of white blood cells into the injured area, generation of reactive oxygen species (ROS), and marked deterioration of muscle's performance [3, 4]. The impressive resemblance of the inflammatory response triggered by the nonbenign, exercise-induced muscle damage and that of muscle trauma render this type of exercise an effective human experimental model to study the mechanisms governing muscle trauma which is present in disease-associated cachexia [5].

Inflammatory leukocytes of myeloid origin, mainly Ly6C^+^/F4/80^−^ neutrophils, start infiltrating the traumatized skeletal muscle tissue immediately after damaging exercise (i.e., eccentric-type exercise) reaching a peak at 6–24 h after injury and then rapidly subsiding [3, 6]. Subsequently, phagocytic CD68^+^/M1 macrophages infiltrate injured muscle at 24–48 h after injury to complete the removal of cellular debris [1, 3, 7]. These myeloid populations of leukocytes trigger the proliferation of myogenic cells via a TNF-*α*- and IL-6-dependent pathway [1, 8]. This response is mediated by proinflammatory cytokines such as IL-1b, TNF-a, IL-6, and IL-8 which are released by muscle and/or leukocytes themselves [9–11] in an NF-kB- and MAPK-dependent induction [12, 13]. Following an attenuation of M1 phagocytic macrophages, CD163^+^/CD206^+^ M2 nonphagocytic macrophages invade the afflicted area in response to the action of antiinflammatory cytokines such as IL-10 and promote muscle growth and repair by stimulating satellite cells [14]. It has been also shown that recovery from strenuous exercise is characterized by an initial rise in total lymphocytes in the circulation and a later accelerated lymphocytopenia [15–17]. This effect has also been seen following muscle-damaging exercise [15, 17], is more noticeable for CD4^+^, CD8^+^ T-cell, and CD3^−^/CD56^+^ natural killer (NK) cell subsets, and has been linked to increased [15, 17, 18] susceptibility of athletes to infections during recovery from exercise [18]. Although the initial increase of circulating lymphocytes has been attributed to an upregulation of their release from lymph nodes or lymphoid organs (e.g., spleen) [17], the later lymphocytopenia may reflect an extravasation of certain lymphocyte subsets from the circulation [17] or may be related to a lymphocyte infiltration into traumatized muscle [15, 19] although the later explanation has been challenged [17, 20]. However, cytotoxic CD8^+^ lymphocytes have been shown to infiltrate injured muscle and demolish nonnecrotic muscle cells in chronic inflammatory diseases [21]. Others have suggested that lymphocytopenia may be related to a reduction of lymphocytes' proliferation, microbicidal activity [22], and increased oxidative DNA damage and apoptosis [23].

ROS produced by leukocytes (due to activation of NADPH oxidase and enzymes such as xanthine oxidase and cyclooxygenase-2) in response to the action of cytokines released by injured muscle as well as by invading neutrophils not only offer antiseptic defence to the muscle but also result in drastic disturbance of muscle's redox status [24–26]. ROS release during postexercise recovery may cause a secondary damage to both afflicted and healthy adjacent myofibers (due to oxidation of muscle's protein and lipid molecules) despite a rise in muscle's antioxidant reserves [11]. Reduced glutathione (GSH) is one of the main antioxidants of muscle and is used to neutralize ROS thereby producing its oxidized form (GSSG), a process that results in marked perturbation of redox status in myofibers [3]. It has been suggested that the GSH/GSSG couple functions as a key-controller of important redox-sensitive intracellular signaling pathways that lead to cytokine synthesis and release by the injured muscle to activate immune cell recruitment and adhesion such as nuclear transcription factor kB (NFkB) and mitogen activated protein kinases (MAPK) [27]. Recent evidence from our group [3] indicated that by attenuating the decline of GSH/GSSG following extensive muscle damage induced by eccentric exercise through administration of a potent thiol-based antioxidant, that is, N-acetylcysteine (NAC), macrophage infiltration in injured muscle, was reduced by ~30% and this response was accompanied by a blunted activation of proinflammatory cytokines and NF-kB and MAPK signaling. Supplementation of antioxidant vitamins did not affect CD4^+^, CD8^+^, naive T cells, NK cells, and proinflammatory cytokines responses following intense eccentric exercise [28]. In contrast, in vivo and in vitro studies have shown that alterations of GSH/GSSG status via administration of NAC may result in reduced neutrophil chemotaxis [29] and macrophage accumulation [30], by attenuating the NF-*κ*B-dependent proinflammatory cytokine expression and release [31, 32], as well as increased lymphocyte numbers and activation by inhibiting their apoptotic rate [33] suggesting that immune responses in inflammatory states may be redox-dependent. However, this possibility has not been explored in humans in inflammation induced by muscle-damaging exercise. Elucidation of this possibility will shed light to the mechanisms involved in skeletal muscle trauma and aid in the development of potential treatments for diseases characterized by muscle inflammation. In this study we utilized NAC supplementation to enhance GSH stores during recovery from a very intense eccentric exercise protocol performed on an isokinetic dynamometer. We hypothesized that NAC administration alters immune cell responses following exercise-induced muscle damage.

## 2. Material and Methods

### 2.1. Study Design

**Figure 1:**
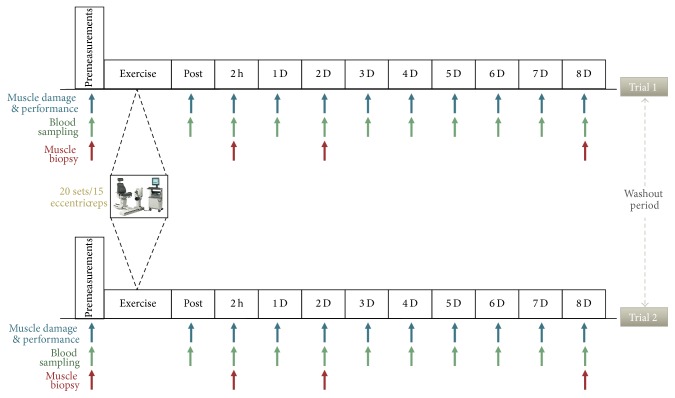
The experimental design of the study. PLA, placebo; NAC, N-acetylcysteine.

In order to determine experimentally whether immune responses to exercise-induced inflammation are redox-sensitive, thiol-based antioxidant supplementation with NAC was utilized according to a cross-over, double-blind, repeated measures design employed in this study. NAC or placebo was administered daily during an 8-day recovery after an acute intense eccentric exercise protocol as previously published [3]. In between trials (NAC and placebo), a 6-week washout phase was utilized. Six weeks have been shown to be adequate for resolution of the inflammatory response induced by this type of eccentric exercise protocol [3]. NAC and placebo trials were administered in a random order for each participant. Prior to each trial, participants had their body weight, height, composition, and cardiovascular conditioning (maximal oxygen consumption, VO_2max_) measured. Blood sampling took place at baseline, immediately after exercise, 2 h after exercise, and daily for eight consecutive days thereafter. Performance (leg strength) and muscle damage markers [delayed onset of muscle soreness (DOMS), knee joint range of motion (KJRM)] were measured at the same time points with blood sampling except immediately after exercise. Testing and blood sampling were administered always at the same time of day. Study's flowchart is shown in Figure 1.

### 2.2. Participants

Ten healthy male volunteers (age, 24.2 ± 2.1 yrs; weight, 78.5 ± 7.8 kg; height, 1.81 ± 0.1 m; body fat, 13.5 ± 3.2%; VO_2max_, 49.2 ± 5.1 mL/kg/min) participated in this study. According to a preliminary power analysis, a sample of 10 participants was necessary to identify noteworthy statistical trial effects among serial measurements following a muscle-damaging protocol at a level of 0.90. Participants' inclusion in the study was decided if (a) they had a VO_2max_ > 45 mL/kg/min, (b) exercised regularly for ≥3 times/week during the last 12 months prior to the study, (c) were nonsmokers, (d) abstained from exercise during the course of the two trials, and (e) did not consume performance-enhancing substances, antioxidants, caffeine, alcohol, and/or medications. Participants were excluded from the study if they had (a) a NAC intolerance and (b) recent musculoskeletal injuries of the lower limbs, febrile illness, and history of muscle lesion. Initially, 22 males were approached and 15 agreed to participate in this investigation. Four volunteers were excluded because they did not fulfil the selection criteria. All subjects provided a signed informed consent form and all experimental procedures were in accordance with the Helsinki Declaration for the ethical treatment of human subjects. Ethics approval was granted by the Institutional Review Board and Ethics Committee.

### 2.3. Exercise Protocol

The exercise protocol included 300 eccentric unilateral repetitions (performed in 20 sets of 15 repetitions/set with a 30 sec rest interval between sets) of knee extensors at a velocity of 30°/sec on an isokinetic dynamometer (Isoforce, TUR Gmbh, Berlin, Germany) as previously published [4]. This protocol has been documented by electron microscopy, biochemistry, and immunohistochemistry to cause extensive microtrauma of muscle fibers of the vastus lateralis [3, 4].

### 2.4. Supplementation Protocol

NAC was ingested orally at a dose of 20 mg NAC/kg/day (Uni-Pharma) in three dosages. Supplementation started immediately after exercise and continued for eight consecutive days thereafter. The supplementation protocol has been described in detail elsewhere [3]. Briefly, NAC was dissolved in water, a sugar-free cordial, and a glucose/dextrose powder. Placebo had the same taste and appearance with NAC. This NAC supplementation protocol has been shown to increase muscle GSH and alter GSH/GSSG ratio [3].

### 2.5. Diet Monitoring and Measurement of Anthropometrics, Cardiovascular Conditioning Skeletal Muscle Performance, and Muscle Damage Markers

Nutritional intake was monitored prior to the first trial using a 7-day diet recall. Diet was also monitored daily during the first trial (1 recall per day). Accordingly, participants were asked to use exactly the same diet before and during the second trial. A registered dietitian trained participants how to complete the diet recalls and records were analyzed with Science Fit Diet 200A (Science Technologies). Body mass and height were measured and body mass index was calculated as previously described [3]. Participants' body composition was measured by Dual Emission X-ray Absorptiometry (DXA) as previously published [34]. VO_2max_ was measured with a gas exchange analyzer (Oxycon Mobile, SensorMedics Corporation) during a graded exercise testing to exhaustion on a treadmill as previously described (Michailidis, 2013). Previous research has shown that cardiovascular and musculoskeletal conditioning level may affect skeletal muscle's antioxidant capacity and redox potential [35, 36]. Muscle performance (maximal knee extensor eccentric peak torque) was measured using an isokinetic dynamometer (Isoforce, TUR Gmbh, Berlin, Germany) at 60°/s as described [4]. The coefficient of variation (CV) for repeated measures was <4%. DOMS was assessed by palpation of the vastus medialis, vastus lateralis, and rectus femoris following a squat motion as previously published [3].

### 2.6. Blood Sampling and Assays

Blood samples (~16 mL) were collected from an antecubital arm vein using a 20-gauge disposable needle with a Vacutainer holder while participants were seated following overnight fasting. Participants refrained from any strenuous physical activity, smoking, and alcohol consumption throughout the experimental period. A blood portion was collected into tubes with ethylenediaminetetraacetic acid (EDTA) that was immediately used for plasma preparation by centrifugation (at 1370 g, 4°C, 15 min) for the measurement of protein carbonyls (PC) and adhesion molecules. Another blood portion was collected into tubes with SST-Gel/clot activator, remained at room temperature for 30 min to clot, and then was used for serum preparation by centrifugation (1500 g, 4°C, 20 min) for the measurement of cytokines, C-reactive protein (CRP), total antioxidant capacity (TAC), and creatine kinase activity (CK). Subsequently, packed red blood cells were collected following disruption of plasma samples according to procedures previously described [37] and the lysates were utilized for the measurement of the concentration of GSH, GSSG, thiobarbituric acid-reactive substances (TBARS), and catalase activity (CAT). Erythrocyte lysates as well as plasma and serum samples were stored at −80°C in multiple aliquots. Another blood portion (2 mL) was collected in EDTA tubes for the assessment of a complete leukocyte count, hematocrit, and hemoglobin on an automated haematology analyser (coulter Beckman LH 750 analyzer, Fullerton, CA, USA) within 2 hours. Light exposure and autooxidation of blood samples were prevented. Samples were analyzed in duplicate.

An enzymatic assay was utilized to measure CK on a Clinical Chemistry Analyzer Z 1145 (Zafiropoulos Diagnostica, Athens, Greece) with a reagent kit (Zafiropoulos P., Greece). CRP and adhesion molecules were measured 2 h after exercise and daily until day 3 of recovery. CRP was assayed using particle-enhanced immunonephelometry (Dade-Behring BN Prospec nephelometer). A multiplex assay kit (Linco, MO, USA) was used to measure the concentration of adhesion molecule sVCAM-1 and sICAM-1 on a Luminex-100 IS (Luminex, USA) as described [38]. Cytokines IL-1b and IL-6 were measured at 2 h after exercise as well as on days 1, 2, 3, and 8 of recovery using a multiple analyte profiling assay (MILLIPLEX Human Cytokine Panel, Millipore Corp) on a Luminex-100 IS Integrated System (Luminex Corporation). GSH was measured spectrophotometrically at 412 nm in TCA-treated erythrocyte lysates as previously published [39]. For the measurement of GSSG, TCA-treated erythrocyte lysates and samples were measured spectrophotometrically at 412 nm for 3 min following procedures as previously described [39]. Calibration curves were constructed for the calculation of GSH and GSSG concentration. TBARS was measured spectrophotometrically (530 nm) as a marker of lipid peroxidation in TCA-treated erythrocyte lysates following addition of thiobarbituric acid as described [39]. PC were assayed spectrophotometrically (375 nm) as an index of protein oxidation in TCA-treated erythrocyte lysates following addition of 2,4-dinitrophenylhydrazine, ethanol–ethyl acetate and urea as described [39]. TAC was measured spectrophotometrically (520 nm) in serum treated with sodium–potassium phosphate and 2,2-diphenyl-1 picrylhydrazyl as described [39]. Spectrophotometric analyses were performed using a Hitachi 2001 UV/VIS (Hitachi Instruments Inc., US) instrument. Inter- and intra-assay CVs for all assays were 2.5–7.3% and 3.7–8.2%, respectively.

Blood samples for flow cytometry were collected into EDTA-coated vacutainer tubes and preserved at room temperature until processed. For isolation of peripheral mononuclear cells (PBMCs), blood samples underwent density gradient centrifugation (400 g, 30 min, room temperature) using Ficoll-paque (Amersham, Uppsala, Sweden) in 15 mL conical tubes. PBMCs were then transferred into a 50 mL conical tube and washed twice with 50 mL of phosphate-buffered salt solution (PBS). Cell number and viability were checked using the trypan blue exclusion test on an improved Neubauer chamber (PRECICOLOR HBG, Germany). Cells were then frozen in freezing solution containing fetal calf serum (FCS) and DMSO (the ratio FCS/DMSO was 1 : 10). Samples were kept at −80°C until various subsets of leukocytes were analyzed by flow cytometry. Prior to analysis by flow cytometry, cells were thawed from −80°C using a 37°C water bath and centrifuged (1,800 rpm, 10 min, room temperature) twice in 50 mL washing buffer (PBS). Cytofluorometric analysis was performed as previously described [40]. Briefly, samples (20 *μ*L) were allocated in tubes containing 100 *μ*L PBS and incubated with a 10 *μ*L antibody solution specific for CD3 FITC-anti-mouse, CD4 PerCP-Cy5,5-anti mouse, CD8 PE-anti mouse, CD14 FITC-anti mouse and CD 14 PEanti mouse, HLA-DR FITC anti-mouse, CD11b PerCP-Cy5,5-anti mouse, CD19 PE-anti mouse, CD45 PerCP-Cy5,5-anti mouse, CD56 PerCP-Cy5,5-anti mouse, and CD62L FITC anti-mouse (all from BD Pharmingen, SanDiego, CA, USA) in the dark for 30 min. Controls used were mouse anti-human IgG isotypes for FITC, PE, and PerCP-Cy5,5. Antigens detected on lymphocytes and monocytes in peripheral blood are shown in Table 1. Samples were then fixed with 400 *μ*L of 1x CellFix solution (BD Pharmingen). Cells underwent a cytofluorometric analysis on a three-colour fluorescence FACSCalibur cytometer and CellQuest software (Becton-Dickinson, San Jose, CA, USA) using laser extinction at 585 nm (phycoerythrin), 530 nm (fluorescein isothiocyanate), and Cy-chrome/PerCP-Cy5.5 695 nm. Cells were detected and electronically gated using forward light-scatter and side light-scatter modes as described [40]. Approximately, 10,000 gated events per condition were analyzed using a standardized gating technique and procedure as described [40]. Values obtained for lymphocyte and monocyte subpopulations represent the percentage of each subset within the overall population of lymphocytes and monocytes, respectively. All samples from a single subject were analyzed in a single run. The typical error of measurement ranged between 2.51% and 4.2%.

### 2.7. Statistical Analysis

Data are presented as means ± SD. Normality was assessed using a Kolmogorov–Smirnov test. A repeated measures ANOVA [trial (placebo and NAC) × time (baseline, after exercise, 2 h after exercise, and 1, 2, 3, 4, 5, 6, 7, and 8 d after exercise or 2 h after exercise, and 1, 2, and 3 d for some inflammatory markers and 2 h after exercise, and 1, 2, and 3 d for cytokines)]. A Bonferonni post hoc test was utilized to determine potential treatment effects. A repeated measures ANOVA (on time) was used to determine potential differences in dependent variables between trials at baseline. Significance was accepted at *P* < 0.05. SPSS software for Windows was used for all statistical analyses (SPSS Inc., Chicago, IL, USA).

## 3. Results

No differences were detected in subjects' body mass, body composition, daily nutrient intake (Table 1), VO_2max_, and leg strength at baseline before each trial. No side effects to NAC consumption were reported by the subjects.

### 3.1. Redox Status Changes

**Figure 2:**
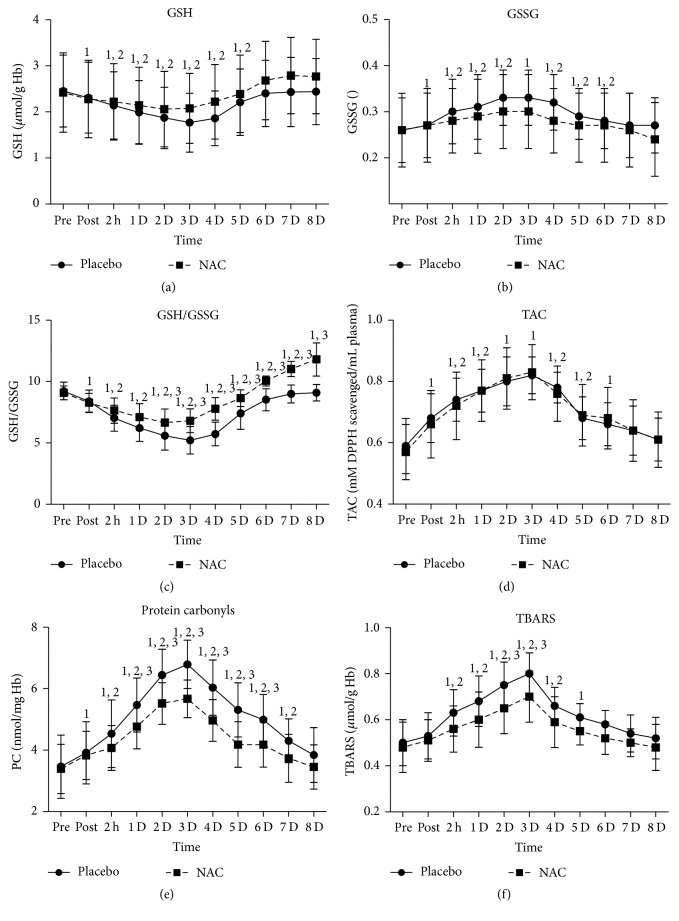
Changes in redox status and oxidative stress markers in response to exercise-induced inflammation. PLA, placebo; NAC, N-acetylcysteine; GSH, reduced glutathione; GSSG, oxidized glutathione; TAC, total antioxidant capacity; PC, protein carbonyls; TBARS, thiobarbituric acid-reactive substances; ^1^significantly different from baseline at *P* < 0.05; ^2^significantly different from the previous time point at *P* < 0.05; ^3^significant difference between trials at *P* < 0.05.

GSH (Figure 2(a)) in PLA was reduced (*P* = 0.000–0.003) immediately after exercise and normalized on day 6 whereas, in NAC, it decreased (*P* = 0.000–0.003) immediately after exercise and normalized on day 5. GSH was maintained 4–16% (*P* = 0.000) higher in NAC compared to PLA throughout recovery (except immediately after exercise). GSSG (Figure 2(b)) increased in PLA immediately after exercise, peaked at 72 h after exercise, remained elevated until day 6 of recovery (3–26%; *P* = 0.000–0.004), and normalized thereafter whereas in NAC it increased after exercise, peaked at 48 h, remained elevated until day 3 of recovery (3–14.5%; *P* = 0.000–0.019), normalized on days 4 through 6, and declined on day 8 (9%; *P* = 0.000). However, no differences were detected between trials. GSH/GSSG ratio (Figure 2(c)) in PLA declined until day 6 of recovery (9–43.5%, *P* = 0.000–0.009), reached its nadir at 48 h, and normalized on days 7 and 8 of recovery whereas in NAC it declined until day 4 (8–26%; *P* = 0.000), reached its nadir at 48 h, normalized on day 5, and increased (10–30%; *P* = 0.000) on days 6 through 8 of recovery. NAC exhibited a smaller decline of GSH/GSSG ratio on days 2 through 5 (*P* = 0.000–0.048) and a greater rise on days 6 through 8 (*P* = 0.000) than PLA. TAC (Figure 2(d)) increased similarly in both trials (PLA: ~17%–41%, *P* = 0.000–0.04; NAC: ~15%–45%, *P* = 0.000–0.02) until day 5 of recovery, peaked on day 3 in both trials, and normalized thereafter. PC (Figure 2(e)) in PLA increased at 2 h and remained elevated throughout recovery, demonstrating a peak on day 3 (11–96%, *P* = 0.000–0.01). PC in NAC increased at 2 h and remained elevated until day 6, demonstrating a peak on day 3 (23–67%, *P* = 0.000–0.06). PC remained higher (*P* = 0.002–0.033) in PLA than NAC in days 1 through 6 of recovery. TBARS (Figure 2(f)) in PLA increased at 2 h and remained elevated until day 5 of recovery, demonstrating a peak on day 3 (22–59%, *P* = 0.000–0.008) whereas in NAC it increased at 2 h and remained elevated until day 4 of recovery, demonstrating a peak on day 3 (16–44%, *P* = 0.000). NAC exhibited lower TBARS levels than PLA on days 2 (*P* = 0.08), 3 (*P* = 0.05), and 6 (*P* = 0.091).

### 3.2. Changes in Muscle Performance and Muscle Damage Markers

**Figure 3:**
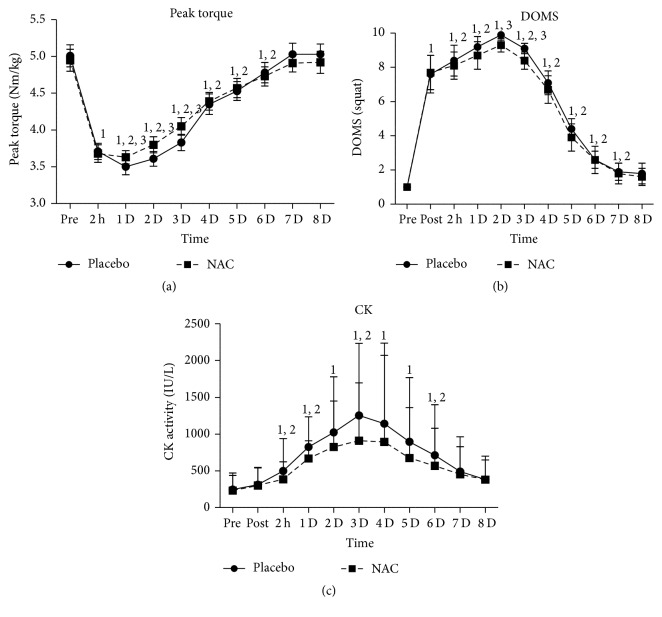
Changes of muscle performance and muscle damage markers. PLA, placebo; NAC, N-acetylcysteine; DOMS, delayed onset of muscle soreness; CK, creatine kinase activity; ^1^significantly different from baseline at *P* < 0.05; ^2^significantly different from the previous time point at *P* < 0.05; ^3^significant difference between trials at *P* < 0.05.

Mean relative (Nm/Kg) eccentric peak torque (Figure 3(a)) declined in both trials (PLA: ~5%–53%, *P* = 0.000; NAC: ~4.5%–43%, *P* = 0.000) 2 h after exercise and during the first six days of recovery reaching both a nadir value on day 2 of recovery. The performance decline in NAC was less pronounced than that observed in PLA during days 1 through 3 of recovery (*P* = 0.001). DOMS (Figure 3(b)) declined in both trials during the first seven days of recovery (PLA: ~1.9–9.9-fold, *P* = 0.000–0.009; NAC: ~1.8–9.3-fold, *P* = 0.000–0.029) reaching its highest value on day 2 for both treatments. PLA induced a greater rise in DOMS than NAC on days 2 (*P* = 0.004) and 3 (*P* = 0.002). CK (Figure 3(c)) in PLA increased at 2 h after exercise (*P* = 0.000), remained elevated (*P* = 0.004–0.039) until day 6 of recovery, and normalized thereafter, reaching its peak on day 3 (~4-fold, *P* = 0.003). CK in NAC increased 1 d after exercise (*P* = 0.001), remained elevated (*P* = 0.021–0.089) until day 4 of recovery, and normalized thereafter, reaching its peak on day 3 (~3-fold, *P* = 0.033). Post hoc analysis revealed no differences between trials throughout recovery.

### 3.3. Changes in Inflammatory Markers

**Figure 4:**
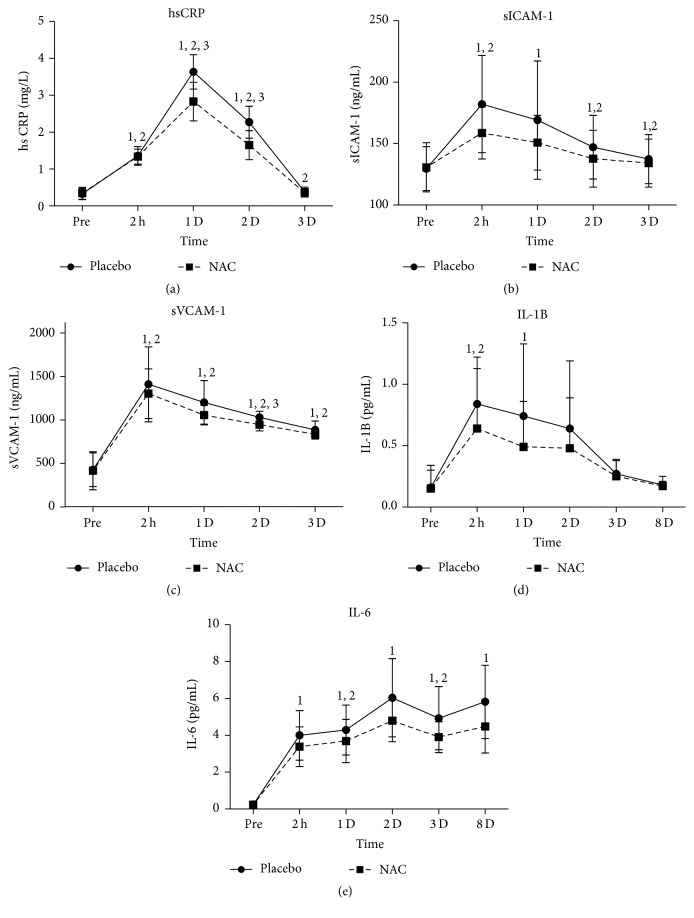
Changes in inflammatory markers. PLA, placebo; NAC, N-acetylcysteine; CRP, C-reactive protein; sICAM-1, soluble intercellular adhesion molecule-1; sVCAM-1, vascular cell adhesion molecule-1; IL-1b, interleukin 1b; IL-6, interleukin 6; ^1^significantly different from baseline at *P* < 0.05; ^2^significantly different from the previous time point at *P* < 0.05; ^3^significant difference between trials at *P* < 0.05.

CRP (Figure 4(a)) remained increased (*P* = 0.000) during the first two days of recovery and normalized thereafter in both trials. PLA demonstrated higher CRP values on days 1 (*P* = 0.002) and 2 (*P* = 0.003) of recovery. sICAM-1 (Figure 4(b)) increased at all time points in PLA (5.5%–40%, *P* = 0.000–0.001) whereas in NAC it decreased only at 2 h after exercise (21%, *P* = 0.005). However, no differences in sICAM-1 changes were noted among trials. sVCAM-1 (Figure 4(c)) declined in both trials (PLA: 1–2.2-fold, *P* = 0.000; NAC: 1–2.1-fold, *P* = 0.000) at all time points measured during recovery with PLA inducing a greater rise (*P* = 0.016) than NAC on day 2 of recovery. IL-1b (Figure 4(d)) increased at 2 h after exercise (3.9-fold, *P* = 0.002) and on day 1 (3.3-fold, *P* = 0.063) in PLA whereas in NAC it increased only at 2 h after exercise (3.2-fold, *P* = 0.021). However, no differences were noted among trials. IL-6 (Figure 4(e)) increased similarly in both trials throughout recovery (PLA: ~15%–23%, *P* = 0.000; NAC: ~13%–18%, *P* = 0.000).

### 3.4. Immune System Responses

**Figure 5:**
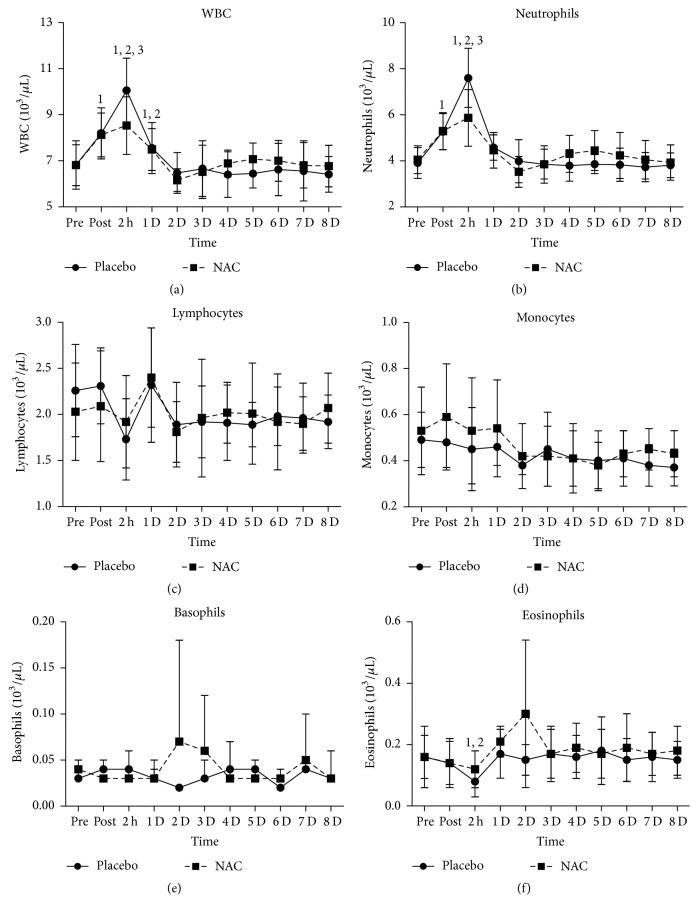
Changes in leukocyte counts. PLA, placebo; NAC, N-acetylcysteine; WBC, white blood cell count; ^1^significantly different from baseline at *P* < 0.05; ^2^significantly different from the previous time point at *P* < 0.05; ^3^significant difference between trials at *P* < 0.05.

White blood cell count (WBC, Figure 5(a)) increased in both trials immediately after exercise (~20%, *P* = 0.000), peaked at 2 h after exercise (PLA: ~47%, *P* = 0.000; NAC: ~25%, *P* = 0.000), remained elevated (PLA: ~11%, *P* = 0.000; NAC: ~10%, *P* = 0.008) 24 h after exercise, and returned to baseline values thereafter. WBC rise was more pronounced (*P* = 0.02) in PLA than NAC at 2 h after exercise. Neutrophil count (Figure 5(b)) increased in both trials after exercise (PLA: ~35%, *P* = 0.000; NAC: ~31%, *P* = 0.000), peaked at 2 h of recovery (PLA: ~95%, *P* = 0.000; NAC: ~45%, *P* = 0.035), and normalized thereafter. PLA demonstrated a greater (*P* = 0.01) rise in neutrophil count than NAC at 2 h after exercise. No time-dependent changes in lymphocyte (Figure 5(c)), monocyte (Figure 5(d)), and basophile (Figure 5(e)) counts were detected in both trials during exercise recovery. Eosinophil count (Figure 5(f)) decreased only (~46%, *P* = 0.011) in PLA at 2 h after exercise.

**Figure 6:**
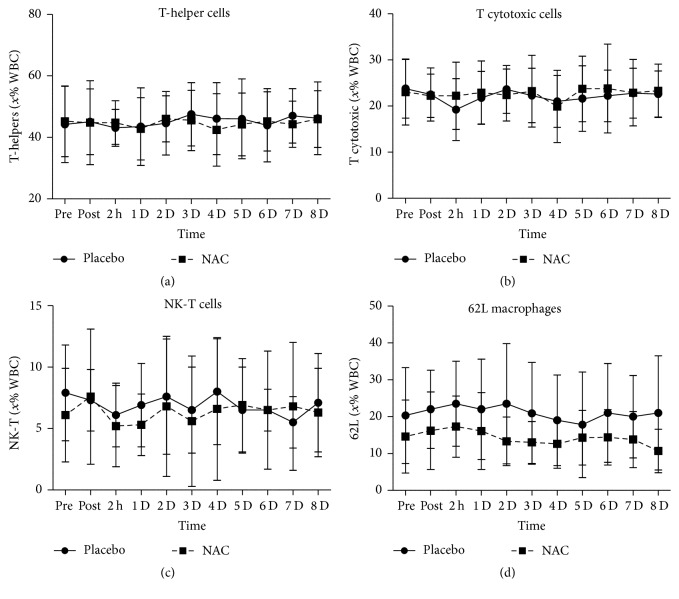
Changes in the levels of T-helper cells, T cytotoxic cells, NK-T cells, and 62L macrophages. PLA, placebo; NAC, N-acetylcysteine; WBC, white blood cell count; ^1^significantly different from baseline at *P* < 0.05; ^2^significantly different from the previous time point at *P* < 0.05; ^3^significant difference between trials at *P* < 0.05.

**Figure 7:**
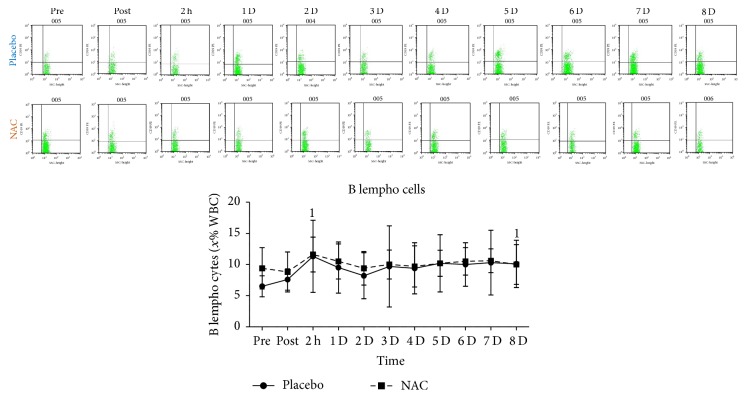
Changes in the levels of B lempho cells. PLA, placebo; NAC, N-acetylcysteine; WBC, white blood cell count; ^1^significantly different from baseline at *P* < 0.05; ^2^significantly different from the previous time point at *P* < 0.05; ^3^significant difference between trials at *P* < 0.05.

**Figure 8:**
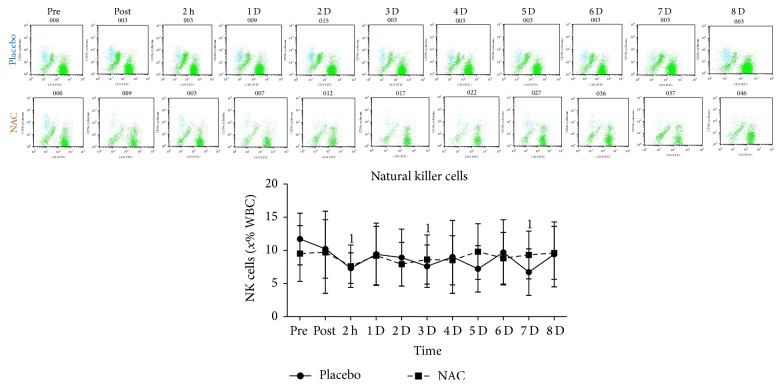
Changes in the levels of natural killer cells. PLA, placebo; NAC, N-acetylcysteine; WBC, white blood cell count; NK, natural killer; ^1^significantly different from baseline at *P* < 0.05; ^2^significantly different from the previous time point at *P* < 0.05; ^3^significant difference between trials at *P* < 0.05.

**Figure 9:**
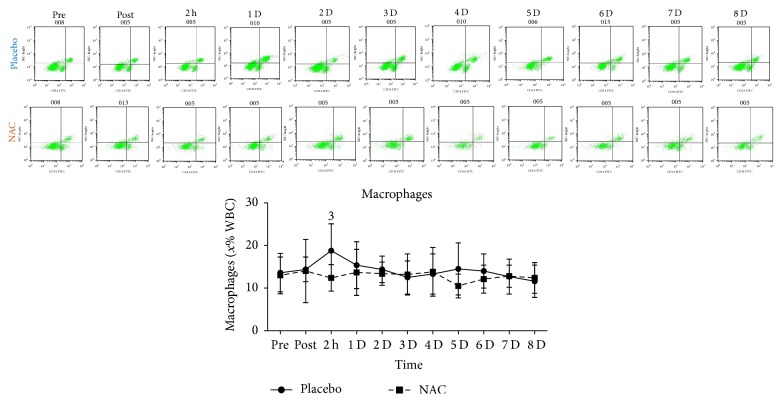
Changes in the levels of macrophages. PLA, placebo; NAC, N-acetylcysteine; WBC, white blood cell count; ^1^significantly different from baseline at *P* < 0.05; ^2^significantly different from the previous time point at *P* < 0.05; ^3^significant difference between trials at *P* < 0.05.

**Figure 10:**
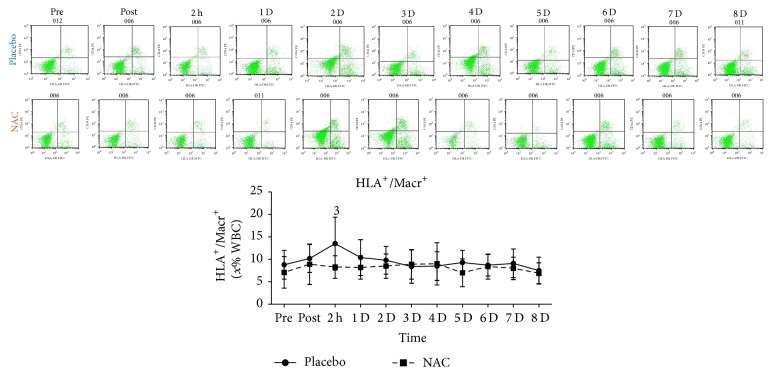
Changes in the levels of HLA^+^/Macr^+^macrophages. PLA, placebo; NAC, N-acetylcysteine; WBC, white blood cell count; ^1^significantly different from baseline at *P* < 0.05; ^2^significantly different from the previous time point at *P* < 0.05; ^3^significant difference between trials at *P* < 0.05.

**Figure 11:**
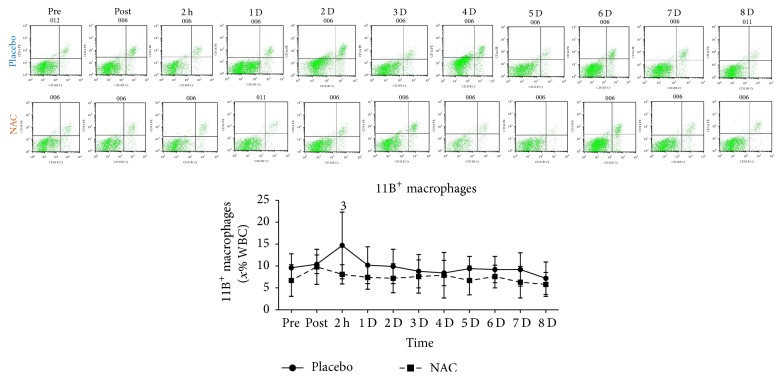
Changes in the levels of 11B^+^ macrophages. PLA, placebo; NAC, N-acetylcysteine; WBC, white blood cell count; ^1^significantly different from baseline at *P* < 0.05; ^2^significantly different from the previous time point at *P* < 0.05; ^3^significant difference between trials at *P* < 0.05.

T-helper cells (Figure 6(a)), T cytotoxic cells (Figure 6(b)), NK-T cells (Figure 6(c)), and 62L macrophages (Figure 6(d)) remained unchanged in both trials throughout recovery. B lympho cells (Figure 7) increased only in PLA 2 h after exercise (68%, *P* = 0.082). Natural killer cells (NK, Figure 8) declined only in PLA 2 h after exercise (~36.5%, *P* = 0.018). When percent changes were compared, NK demonstrated a greater decline in PLA than NAC 2 h after exercise (~36.5% in PLA versus ~12% in NAC, *P* = 0.09) as well as at 3 d (~35% in PLA versus ~3% in NAC, *P* = 0.036), 5 d (~35% in PLA versus ~19% in NAC, *P* = 0.062), and 7 d (~42% in PLA versus ~14% in NAC, *P* = 0.039). Although macrophages (Figure 9) demonstrated no time-dependent changes in both trials, statistical analysis revealed that PLA induced a more pronounced rise than NAC 2 h after exercise (~53% in PLA versus ~1% in NAC, *P* = 0.016). Likewise, HLA^+^/Macr^+^ (Figure 10) and 11B+ macrophages (Figure 11) exhibited no time-dependent changes in both groups throughout recovery but statistical analysis revealed that PLA demonstrated a greater increase than NAC 2 h after exercise (HLA^+^/Macr^+^: ~75% in PLA versus ~48% in NAC, *P* = 0.027; 11B+: ~66.5% in PLA versus ~39.5% in NAC, *P* = 0.025).

## 4. Discussion

The present investigation provides evidence that mobilisation of leukocytes in response to exercise-induced inflammation induced by very intense exercise may be redox dependent. These results verify previous observations of a redox-sensitive regulation of exercise-induced inflammation and muscle performance [3, 41].

NAC ingestion protocols similar to ours have been shown to increase NAC bioavailability in the circulation [42] probably due to a reduction of its clearance from the circulation during exercise [43]. As previously shown under conditions of elevated oxidative stress [3, 44, 45], NAC reduced (4–16%) the decline of GSH during the first five days of recovery and maintained it at a higher level than PLA on days 6–8 of recovery (~10–15%). Similar responses of erythrocyte GSH have been reported under in vivo and in vitro conditions [46, 47]. It appears that NAC supplementation does not affect GSH resynthesis from GSSG but it rather upregulates GSH availability thereby altering GSH/GSSG due to a rise in intracellular cysteine concentration due to its increased active transport from extracellular space using the g-glutamyl cycle, at least in muscle and erythrocytes [35, 45–48]. A single oral bolus of 600 mg NAC to humans raises free reduced NAC concentration in plasma at ~2.5–4.6 uM within 60–90 min verifying the relatively fast uptake of NAC by blood cells such as the 60-minute uptake by erythrocytes [46] and implies that NAC effects on these cells are mediated mostly intracellularly rather than by an upregulation of extracellular antioxidant environment [49]. These findings are in agreement with the GSH rise seen within the first 2 h after exercise in this study.

Under stress, NAC consumption may not affect total GSH levels but rather causes an attenuation of GSH decline because it increases free sulfhydryl groups in the circulation [36, 45, 46, 50–52] resulting in an upregulation of intracellular NAC levels and free cysteine residues by rapid deacetylation which then elevate intracellular GSH and alter GSH/GSSG [35, 45, 53]. ROS scavengers may promote GSH sparing and thus lessen GSH depletion and GSH/GSSG reduction (redox status) intracellularly as seen in muscle, erythrocytes, and leukocytes [3, 36, 41, 45, 54]. Postexercise GSH sparing and accelerated normalization of redox status in response to NAC ingestion has also been observed extracellularly, that is, in the circulation [55, 56]. GSH may further contribute to the attenuation of GSH/GSSG decline and the blunted elevation of oxidative stress markers (i.e., TBARS, PC) via the glutathione peroxidase reaction that mediates H_2_O_2_ clearance [35]. NAC administration not only accelerates the normalization of GSH and GSH/GSSH after exercise but also mitigates muscle performance drop during recovery, as in this study [3, 41, 57]. NAC may also enhance the GSH-dependent estrogenic activity which contributes to induction of genes encoding enzymatic proteins regulating GSH turnover such as GSH reductase [58]. Redox changes may regulate redox-sensitive intracellular signaling pathways that control cellular phenotype under stress [3, 41]. However, at basal state, when oxidative stress is normalized, NAC ingestion may not alter oxidative stress markers despite a rise in GSH and GSH/GSSG [59, 60] as shown in days 5–8 of postexercise recovery in this study.

The eccentric exercise protocol used in this study has been shown to cause a marked skeletal muscle damage as documented by both histochemical and biochemical data [3, 4]. The pronounced rise of CK after exercise was accompanied by an intense acute inflammatory response (marked rise of WBC, CRP, cytokines, and adhesion molecules) and a substantial decline of muscle performance in both trials. The reduced muscle damage response in NAC may be further explained by previous observations of attenuated eccentric exercise-induced muscle injury following neutrophil and macrophage depletion suggesting that neutrophil's/macrophages' oxidative burst may be responsible for part of muscle's injury during early recovery [32, 61]. Therefore, the blunted rise of neutrophils in NAC may partly explain the attenuated muscle damage (blunted CK and CRP rise). Muscle function was better protected by NAC during that period, an effect reported by previous studies [35, 62, 63]. This protective effect of NC may be related to GSH enhancement. GSH deficiency has been shown to impair muscle function [56]. NAC may be able to maintain the activity of Na+/K+ pump in muscle due to a redox-dependent [64, 65] attenuation of sarcoplasmic reticulum injury [11] that results in a lower calcium release and thus a reduced activity of muscle's proteolytic proteins such as the proteosome and calpains [66, 67].

### 4.1. Neutrophil Responses

Exercise-induced muscle injury triggers a pronounced immune response which is expressed as an early (≤24 h) mobilization and infiltration of neutrophils into traumatized tissue, followed by a later activation and entry of macrophages [1, 3, 11]. This immune mobilization was also observed in this study. The rise in neutrophil count in peripheral blood is caused by exercise-induced demargination [62]. When neutrophils reach the injured tissue using a process called margination, they remove cellular debris using phagocytosis and discharge proteolytic enzymes and ROS into the extracellular space and into the phagosome [11]. ROS release is achieved through a process known as respiratory burst during which activated neutrophils and other phagocytes generate increased numbers of superoxide anions which then are converted to powerful oxidants (i.e., H_2_O_2_ and hypochloride) [68]. This process may cause secondary damage to healthy neighboring tissues [68]. A decline in respiratory burst has also been seen in healthy volunteers following a 14 d NAC ingestion [49]. NAC-induced attenuation of neutrophils' oxidative burst has been consistently seen in animal tissues as well [69] and it may be attributed to a reduction of myeloperoxidase activity (MPO) [70] and/or cellular ATP [49]. A reduction of MPO could be explained by a direct scavenging action by NAC on ROS produced by MPO, or by noncompetitive mechanism by binding to MPO or even by competitive inhibition at or near the active site of the enzyme by GSH [71]. The NAC-related decline of cellular ATP may be associated with an inhibition of sodium ions due to altered activity of sodium channels independent of an effect on Na+–K+-ATPase activity and K+ conductance resulting in increased levels of sodium intracellularly and leakage of potassium ions into the extracellular space [72]. This ion exchange results in entry of water into the cell, expansion of endoplasmic reticulum, and injury to ribosomes that stops protein synthesis [72].

The rise of WBC and neutrophil count was attenuated by NAC administration during the first postexercise hours. Although not statistically significant, it is physiologically interesting that neutrophils remained more elevated (~12%) in PLA compared to NAC 1 d after exercise. In contrast with our results, GSH and NAC supplementation in rats augmented neutrophil mobilization and respiratory burst and reduced leukocyte margination suggesting a thiol-induced upregulation of neutrophils after exercise [62]. However, the majority of human, animal, and in vitro studies are in agreement with results of this study. Supplementation with sulfur-containing amino acids (cysteine, theanine) also led to increased GSH and an attenuated neutrophilic response to very intense exercise [73]. Similarly, pretreatment of patients with chronic obstructive pulmonary disease with NAC upregulated thiol levels and effectively reduced neutrophil chemotaxis and ROS [29]. Similarly, high dosages (0.6–1.0 g) of NAC administered orally three times daily for 4 weeks to patients with cystic fibrosis increased neutrophil GSH levels and reduced airway inflammation (neutrophil migration in airways, elastase activity, and IL-8 levels) [71]. NAC administration to older females, neutrophil function (adherence, chemotaxis, phagocytosis, and oxidative burst), and oxidative stress were normalized and approached values of younger females further supporting a redox-dependent regulation of neutrophil function [54]. NAC infusion to rats with sepsis resulted in reduced oxidative stress, neutrophil density, and IL-1b [30, 70, 74]. Similarly, neutrophil function was inhibited by a 3% NAC solution in mares and mitigated the ROS-mediated injury to the endometrial epithelium and sperm by attenuating neutrophils' oxidative burst [69].

NAC's antineutrophilic action may be mediated by several mechanisms. NAC-induced changes in redox status seem to blunt the rise of proinflammatory cytokines such as IL-1b, IL-6, and IL-8 as seen in this study [3, 29, 74] probably due to an inhibition of their gene expression and/or protein synthesis [3, 32]. Synthesis and release of proinflammatory cytokines are directly or indirectly dependent on NF-kB phosphorylation and p38MAPK activation which are also under redox-sensitive regulation and are subjected to downregulation by NAC for several days after exercise [1, 3, 12, 13]. NAC-induced suppression of proinflammatory cytokines may result in compromised neutrophil survival. Neutrophilia observed during early inflammation is terminated by neutrophil apoptosis and their removal by macrophages in an attempt to minimize oxidative damage. This process is however delayed by proinflammatory chemokines and cytokines [75] and thus prevent engulfment of neutrophils by macrophages [76]. NAC on the other hand may promote neutrophil apoptosis and removal by increasing neutrophil GSH thereby leading to altered redox status and reduction of expression and release of proinflammatory cytokines such as IL-1b and TNF*α* [3, 29, 74] as well as by promoting chemotaxis of phagocytes [54].

A NAC-related blunted rise of proinflammatory cytokines may also affect neutrophil migration and extravasation [77]. For example, sICAM-1, an adhesion molecule mediating neutrophil transmigration, is downregulated due to NAC-induced inhibition of TNF-a, IL-1b, and IL-8 expression [78]. Inhibited neutrophil migration may also result in a NAC-related decline in IL-8 dependent elastase release by neutrophils [79] and facilitate the downregulation of molecules released by the endothelial epithelial bilayer that promote neutrophil migration [49]. For instance, inhibition of redox-dependent MAPK signaling by NAC [3] disrupts LTB4 signaling that facilitates transepithelial migration via a redox-dependent interaction of adhesion molecules with neutrophils and proteins [80]. NAC has also been shown to inhibit sVCAM-1 expression by downregulating NF-*κ*B binding to its gene motif and thus reducing neutrophils' adhesion to endothelium and migration into the affected tissue [81]. In fact, sICAM-1 and sVCAM-1 in NAC demonstrated a blunted response to exercise in this study. These results indicate a redox-dependent control of neutrophils' kinetics and function in response to exercise-induced injury.

### 4.2. Macrophage Responses

Phagocytic macrophages infiltrate the endomysium and perimysium of injured skeletal muscle after neutrophil invasion reaching a peak 1 d after exercise and remain elevated for ~2–4 d depending on the magnitude of the insult [1, 3, 9]. Macrophages of M1 phenotype are proinflammatory and contribute to propagation of inflammation by producing cytotoxic nitric oxide and thus inducing further muscle damage [1]. M1 macrophages, after their peak, are usually replaced by M2 macrophages that promote resolution of inflammation and repair of injured tissue [1]. In this study, although statistically nonsignificant, an increase was observed in total macrophages (4–53% in PLA, 1–14% in NAC) as well as in HLA^+^/Macr^+^ (4–75% in PLA, 8–53% in NAC) and 11B+ macrophages (up to 66% in PLA, up to 70% in NAC) during recovery.

Michailidis et al. [3] reported a dampened response for 68^+^ macrophage infiltration in injured muscle using the same exercise protocol. Similarly, our results revealed that total macrophages (~53% in PLA versus ~1% in NAC), HLA^+^/Macr^+^ (75% in PLA versus 48% in NAC), and 11B^+^ (66% in PLA versus 40% in NAC) response to exercise was attenuated in NAC compared to PLA at 2 h after exercise. Although data from exercise studies is scarce, attenuation of macrophage response has been shown by in vivo and in vitro nonexercise studies. NAC appears to increase intracellular GSH and alter redox status of macrophages [32, 82, 83]. NAC ingestion to humans caused a marked reduction in macrophage number as well as in IL-1b and IL-6 concentration during an ischemia-reperfusion model [74]. Furthermore, in ApoE^−/−^ mice, NAC consumption reduced ROS production, VCAM-1 synthesis, and accumulation of macrophages in atherosclerotic plaque [84]. In vitro results revealed that NAC administration resulted in a redox-dependent reduction in macrophage survival [85] and PBMC proliferation [86].

ROS generation by the NADPH oxidase complex is probably required for macrophage survival since inhibition of this process leads to reduced survival rates in a redox-dependent manner and is associated with an attenuation of Akt and p38 MAPK signaling [85]. NAC consumption during eccentric exercise-induced inflammation has been shown to attenuate both Akt/mTOR and p38 MAPK signaling [3]. According to Geudens et al. [74], IL-6 produced by immune cells in response to IL-1b activation may exert a pleiotropic action by further increasing macrophage mobilisation under inflammatory load, a response that is consistently blunted by NAC under such conditions [3, 32, 74, 86]. This attenuation of cytokine response by NAC may occur at posttranscriptional level [32] and is NF-*κ*B-dependent [3, 74, 84]. Others suggested that NAC at high dosages may promote phagocytosis and intracellular killing in alveolar macrophages indicating that NAC may induce self-killing of macrophages [87]. Furthermore, NAC may interfere with adherence and chemotaxis of macrophages under inflammatory conditions [83, 84]. Collectively, these finding suggest that macrophage mobilisation under inflammatory conditions induced by exercise-induced microtrauma may be redox-dependent.

### 4.3. Lymphocytes' Responses

Although lymphocyte count, T-helper cells, T cytotoxic cells, and NK-T cells remained unaffected by exercise in both trials, B lympho cells (a lymphocyte subtype that expresses clonally diverse cell surface immunoglobulin receptors recognizing specific antigenic epitopes) increased only in PLA 2 h after exercise and natural killer (NK) cells declined only in PLA 2 h after exercise. Intense exercise-induced lymphocytopenia and immunosuppression is a well-described phenomenon [88]. High-intensity running results in a considerable apoptosis of lymphocytes within 24 h of recovery [88, 89]. In line with our results, Nielsen et al. [90] also demonstrated a reduction of NK cell activity and proliferation within 2 h of exercise recovery [90]. This exercise-induced reduction of NK may be attributed to ROS that increase peroxidation levels of lipids located in their membranes [91, 92]. Similarly, Petersen et al. [41] showed that intense exercise results in a fall of lymphocyte levels during recovery. Exercise-induced lymphocytopenia (~40% reduction in lymphocyte levels within the first 24 h of exercise recovery) may be attributed to increased apoptosis due to mitochondrial membrane depolarization, increased ROS, and a decline in intracellular GSH [88, 93]. Intense prolonged running has been shown to elicit DNA base oxidation in human leukocytes that was correlated with lipid hydroperoxides levels in the circulation [94]. Moreover, increased blood isoprostanes may be adequate to promote lymphocyte apoptosis [89, 95]. This is further supported by observations of reduced lymphocyte DNA fragmentation by antioxidant supplementation [96, 97] which is in line with our findings of an abolishment of lymphocyte fall after exercise in NAC.

Supplementation with sulfur-containing amino acids (cysteine, theanine) also attenuated exercise-induced lymphocytopenia [73]. GSH upregulation may inhibit, at least partially, lymphocyte membrane depolarization and DNA oxidation [98, 99]. In fact, GSH-depleted thymocytes were more susceptible to apoptosis [100] whereas NAC appears to inhibit GSH depletion and enhance lymphocyte survival [88]. Furthermore, NAC reduced lymphocyte apoptosis due to exposure to oxidative stress related apoptotic insults (glucocorticoids, H_2_O_2_, Fas, and TCR signaling) [93, 101, 102]. In fact, NAC appears to reduce lymphocyte apoptosis through a mitigation of the rise of proapoptotic proteins (caspase 3 and cytosolic cytochrome c) and an elevation of antiapoptotic proteins (Bcl-2) after exercise [33].

Apoptotic lymphocytes seem to express elevated levels of Fas and FasL 24 h after heavy physical exertion [93, 103]. NF-*κ*B that mediates the communication between ROS and Fas signaling is activated and transported into the nucleus rapidly in response to exercise-induced muscle injury and induces the synthesis of proinflammatory cytokines [3, 104]. NF-*κ*B downregulation by NAC may also explain the attenuated lymphocyte apoptosis by thiol-based antioxidants. Interestingly, exercise training results in improved GSH metabolism and antioxidant capacity and reduced TNF*α*-induced apoptosis [105]. Treatment of cells with thiol-based antioxidants (GSH, cysteine, and NAC) abolishes NF-*κ*B activation [26, 41, 106] due to an inhibition of TNFa-induced activation of NF-*κ*B and specifically to an attenuation of TNFa binding to its receptor [107]. Moreover, NAC pretreatment of macrophages was able to prevent a rise of free p50/p65 heterodimers following exposure to ionizing radiation [108]. In fact, NAC has been shown to inhibit the transfer of p65 protein to the nucleus from cytoplasm in piglets [109]. In line with these observations, NAC inhibited IKKa/b and IkBa phosphorylation and thus the stimulation of NF-*κ*B in endothelial cells following exposure to silver nanoparticles [110]. Other sulfur-containing antioxidants such as pyrrolidine dithiocarbamate have also been shown to inhibit NF-*κ*B after exercise due to a partial reduction of nuclear NF-kB binding, a complete inhibition of IkBa phosphorylation, and degradation and a rise of p50 concentration in the nucleus [111]. In general, antioxidants are capable of reducing peroxide-stimulated NF-*κ*B activity [112]. These effects of thiol-based antioxidants are coupled with blunting of NF-*κ*B transcriptional activity [13].

In contrast, Nielsen et al. [90] reported that the impaired NK cell activity and mitogen-stimulated lymphocyte proliferation were not abolished by NAC after exercise and failure of antioxidant supplements to attenuate exercise-induced lymphocytopenia was not prevented when other antioxidants were used. A cocktail of vitamins C and E failed to observe changes in exercise-induced changes of lymphocyte levels [41]. Furthermore, immune responses following a 2.5 h run remained unaffected by vitamin C supplementation [113]. Contradictory results reported by these studies may be attributed to differences in exercise protocols used, the amount of muscle mass involved, and the degree of induced inflammation and lymphocyte apoptosis. For example, Nielsen et al. [90] used a 6-minute “all-out” ergometer rowing which is different than a 300-repetition eccentric exercise protocol in terms of metabolic requirements and degree of induced muscle damage and inflammatory responses.

In conclusion, our data provide evidence of a redox-dependent regulation of immune responses to skeletal muscle damage induced by intense eccentric exercise. The attenuation of immune responses following NAC consumption seems to be related to an enhancement of GSH levels that lead to redox status changes which ultimately results in changes of proinflammatory cytokines and adhesion molecules.

## Figures and Tables

**Table 1:** Participants' characteristics during the study.

	Placebo trial	NAC trial
Age (years)	24.6 ± 2.5	24.9 ± 2.5
Body mass (kg)	79.0 ± 7.3	79.2 ± 7.1
Body height (m)	1.81 ± 0.1	1.81 ± 0.1
BMI (kg/m^2^)	24.0 ± 0.4	24.1 ± 0.4
Body fat (%)	15.9 ± 2.3	15.8 ± 2.1
VO_2max_ (mL/kg/min)	50.4 ± 3.9	50.6 ± 3.6
Total daily energy intake (kcal)	2,782.2 ± 255.0	2,764.2 ± 237.8
Carbohydrate daily intake (%)^1^	59.4 ± 3.2	59.1 ± 2.8
Fat daily intake (%)^1^	24.8 ± 1.1	24.6 ± 1.0
Protein daily intake (%)^1^	15.8 ± 3.5	16.2 ± 3.0
Selenium (mg/d)	47.5 ± 3.3	47.2 ± 3.2
Zinc (mg/d)	13.5 ± 2.0	13.9 ± 1.2
Vitamin C (mg/d)	132.3 ± 9.8	131.8 ± 8.5
Vitamin E (mg/d)^2^	9.2 ± 1.1	9.3 ± 0.9

Values are presented as means ± SDs. NAC, N-acetylcysteine; BMI, body mass index; VO_2max_, maximal oxygen consumption; ^1^percent of total daily energy intake; ^2^a-tocopherol equivalents.

## References

[1] Tidball J. G., Villalta S. A. (2010). Regulatory interactions between muscle and the immune system during muscle regeneration. *American Journal of Physiology—Regulatory Integrative and Comparative Physiology*.

[2] Smith C., Kruger M. J., Smith R. M., Myburgh K. H. (2008). The inflammatory response to skeletal muscle injury: illuminating complexities. *Sports Medicine*.

[3] Michailidis Y., Karagounis L. G., Terzis G. (2013). Thiol-based antioxidant supplementation alters human skeletal muscle signaling and attenuates its inflammatory response and recovery after intense eccentric exercise. *The American Journal of Clinical Nutrition*.

[4] Raastad T., Owe S. G., Paulsen G. (2010). Changes in calpain activity, muscle structure, and function after eccentric exercise. *Medicine and Science in Sports and Exercise*.

[5] Fehrenbach E., Schneider M. E. (2006). Trauma-induced systemic inflammatory response versus exercise-induced immunomodulatory effects. *Sports Medicine*.

[6] Fielding R. A., Manfredi T. J., Ding W., Fiatarone M. A., Evans W. J., Cannon J. G. (1993). Acute phase response in exercise. III. Neutrophil and IL-1 beta accumulation in skeletal muscle. *The American Journal of Physiology*.

[7] St Pierre B. A., Tidball J. G. (1994). Differential response of macrophage subpopulations to soleus muscle reloading after rat hindlimb suspension. *Journal of Applied Physiology*.

[8] Pavlath G. K., Dominov J. A., Kegley K. M., Millert J. B. (2003). Regeneration of transgenic skeletal muscles with altered timing of expression of the basic helix-loop-helix muscle regulatory factor MRF4. *American Journal of Pathology*.

[9] Paulsen G., Mikkelsen U. R., Raastad T., Peake J. M. (2012). Leucocytes, cytokines and satellite cells: what role do they play in muscle damage and regeneration following eccentric exercise?. *Exercise Immunology Review*.

[10] Isanejad A., Saraf Z. H., Mahdavi M., Gharakhanlou R., Shamsi M. M., Paulsen G. (2015). The effect of endurance training and downhill running on the expression of IL-1*β*, IL-6, and TNF-*α* and HSP72 in rat skeletal muscle. *Cytokine*.

[11] Peake J., Nosaka K., Suzuki K. (2005). Characterization of inflammatory responses to eccentric exercise in humans. *Exercise Immunology Review*.

[12] Sigala I., Zacharatos P., Toumpanakis D. (2011). MAPKs and NF-*κ*B differentially regulate cytokine expression in the diaphragm in response to resistive breathing: the role of oxidative stress. *American Journal of Physiology—Regulatory Integrative and Comparative Physiology*.

[13] Vlahopoulos S., Boldogh I., Casola A., Brasier A. R. (1999). Nuclear factor-*κ*B-dependent induction of interleukin-8 gene expression by tumor necrosis factor *α*: evidence for an antioxidant sensitive activating pathway distinct from nuclear translocation. *Blood*.

[14] Vezzoli M., Castellani P., Campana L. (2010). Redox remodeling: a candidate regulator of HMGB1 function in injured skeletal muscle. *Annals of the New York Academy of Sciences*.

[15] Pizza F. X., Mitchell J. B., Davis B. H., Starling R. D., Holtz R. W., Bigelow N. (1995). Exercise-induced muscle damage: effect on circulating leukocyte and lymphocyte subsets. *Medicine and Science in Sports and Exercise*.

[16] Nieman D. C., Simondie S., Henson D. A. (1995). Lymphocyte proliferative response to 2.5 hours of running. *International Journal of Sports Medicine*.

[17] Simpson R. J., Florida-James G. D., Whyte G. P., Guy Z. K. (2006). The effects of intensive, moderate and downhill treadmill running on human blood lymphocytes expressing the adhesion/activation molecules CD54 (ICAM-1), CD18 (*β*
_2_ integrin) and CD53. *European Journal of Applied Physiology*.

[18] Pedersen B. K., Ullum H. (1994). NK cell response to physical activity: possible mechanisms of action. *Medicine and Science in Sports and Exercise*.

[19] Round J. M., Jones D. A., Cambridge G. (1987). Cellular infiltrates in human skeletal muscle: exercise induced damage as a model for inflammatory muscle disease?. *Journal of the Neurological Sciences*.

[20] Malm C., Nyberg P., Engström M. (2000). Immunological changes in human skeletal muscle and blood after eccentric exercise and multiple biopsies. *The Journal of Physiology*.

[21] Engel A. G., Arahata K. (1986). Mononuclear cells in myopathies: quantitation of functionally distinct subsets, recognition of antigen-specific cell-mediated cytotoxicity in some diseases, and implications for the pathogenesis of the different inflammatory myopathies. *Human Pathology*.

[22] Nieman D. C. (1994). Exercise, upper respiratory tract infection, and the immune system. *Medicine and Science in Sports and Exercise*.

[23] Tanimura Y., Shimizu K., Tanabe K., Kono I., Ajisaka R. (2010). Effects of three consecutive days exercise on lymphocyte DNA damage in young men. *European Journal of Applied Physiology*.

[24] Gomez-Cabrera M.-C., Borrás C., Pallardo F. V., Sastre J., Ji L. L., Viña J. (2005). Decreasing xanthine oxidase-mediated oxidative stress prevents useful cellular adaptations to exercise in rats. *The Journal of Physiology*.

[25] Nikolaidis M. G., Jamurtas A. Z., Paschalis V., Fatouros I. G., Koutedakis Y., Kouretas D. (2008). The effect of muscle-damaging exercise on blood and skeletal muscle oxidative stress: magnitude and time-course considerations. *Sports Medicine*.

[26] Ji L. L. (2007). Antioxidant signaling in skeletal muscle. *Experimental Gerontology*.

[27] Zhou L. Z.-H., Johnson A. P., Rando T. A. (2001). NF*κ*B and AP-1 mediate transcriptional responses to oxidative stress in skeletal muscle cells. *Free Radical Biology and Medicine*.

[28] Petersen E. W., Ostrowski K., Ibfelt T. (2001). Effect of vitamin supplementation on cytokine response and on muscle damage after strenuous exercise. *American Journal of Physiology-Cell Physiology*.

[29] Milara J., Juan G., Peiró T., Serrano A., Cortijo J. (2012). Neutrophil activation in severe, early-onset COPD patients versus healthy non-smoker subjects in vitro: effects of antioxidant therapy. *Respiration*.

[30] Campos R., Shimizu M. H. M., Volpini R. A. (2012). N-acetylcysteine prevents pulmonary edema and acute kidney injury in rats with sepsis submitted to mechanical ventilation. *American Journal of Physiology—Lung Cellular and Molecular Physiology*.

[31] Tsai H.-H., Lee W.-R., Wang P.-H., Cheng K.-T., Chen Y.-C., Shen S.-C. (2013). Propionibacterium acnes-induced iNOS and COX-2 protein expression via ROS-dependent NF-*κ*B and AP-1 activation in macrophages. *Journal of Dermatological Science*.

[32] Palacio J. R., Markert U. R., Martínez P. (2011). Anti-inflammatory properties of N-acetylcysteine on lipopolysaccharide- activated macrophages. *Inflammation Research*.

[33] Quadrilatero J., Hoffman-Goetz L. (2005). N-Acetyl-L-Cysteine inhibits exercise-induced lymphocyte apoptotic protein alterations. *Medicine and Science in Sports and Exercise*.

[34] Terzis G., Stratakos G., Manta P., Georgiadis G. (2008). Throwing performance after resistance training and detraining. *Journal of Strength and Conditioning Research*.

[35] Sen C. K., Packer L. (2000). Thiol homeostasis and supplements in physical exercise. *American Journal of Clinical Nutrition*.

[36] Medved I., Brown M. J., Bjorksten A. R., Leppik J. A., Sostaric S., McKenna M. J. (2003). N-acetylcysteine infusion alters blood redox status but not time to fatigue during intense exercise in humans. *Journal of Applied Physiology*.

[37] Barbas I., Fatouros I. G., Douroudos I. I. (2011). Physiological and performance adaptations of elite Greco-Roman wrestlers during a one-day tournament. *European Journal of Applied Physiology*.

[38] Mohr M., Draganidis D., Chatzinikolaou A. (2016). Muscle damage, inflammatory, immune and performance responses to three football games in 1 week in competitive male players. *European Journal of Applied Physiology*.

[39] Theodorou A. A., Nikolaidis M. G., Paschalis V. (2010). Comparison between glucose-6-phosphate dehydrogenase-deficient and normal individuals after eccentric exercise. *Medicine and Science in Sports and Exercise*.

[40] Sorichter S., Martin M., Julius P. (2006). Effects of unaccustomed and accustomed exercise on the immune response in runners. *Medicine and Science in Sports and Exercise*.

[41] Petersen A. C., McKenna M. J., Medved I. (2012). Infusion with the antioxidant N-acetylcysteine attenuates early adaptive responses to exercise in human skeletal muscle. *Acta Physiologica*.

[42] Borgström L., Kågedal B. (1990). Dose dependent pharmacokinetics *N*-acetylcysteine after oral dosing to man. *Biopharmaceutics and Drug Disposition*.

[43] Brown M., Bjorksten A., Medved I., McKenna M. (2004). Pharmacokinetics of intravenous N-acetylcysteine in men at rest and during exercise. *European Journal of Clinical Pharmacology*.

[44] Sen C. K., Marin E., Kretzschmar M., Hanninen O. (1992). Skeletal muscle and liver glutathione homeostasis in response to training, exercise, and immobilization. *Journal of Applied Physiology*.

[45] Medved I., Brown M. J., Bjorksten A. R. (2004). N-acetylcysteine enhances muscle cysteine and glutathione availability and attenuates fatigue during prolonged exercise in endurance-trained individuals. *Journal of Applied Physiology*.

[46] Matuszczak Y., Farid M., Jones J. (2005). Effects of N-acetylcysteine on glutathione oxidation and fatigue during handgrip exercise. *Muscle and Nerve*.

[47] Lee R., Britz-McKibbin P. (2009). Differential rates of glutathione oxidation for assessment of cellular redox status and antioxidant capacity by capillary electrophoresis-mass spectrometry: an elusive biomarker of oxidative stress. *Analytical Chemistry*.

[48] Atalay M., Marnila P., Lilius E., Hanninen O., Sen C. K. (1996). Glutathione-dependent modulation of exhausting exercise-induced changes in neutrophil function of rats. *European Journal of Applied Physiology and Occupational Physiology*.

[49] Sadowska A. M., Manuel-Y-Keenoy B., Vertongen T. (2006). Effect of N-acetylcysteine on neutrophil activation markers in healthy volunteers: in vivo and in vitro study. *Pharmacological Research*.

[50] Merry T. L., Wadley G. D., Stathis C. G. (2010). N-Acetylcysteine infusion does not affect glucose disposal during prolonged moderate-intensity exercise in humans. *Journal of Physiology*.

[51] Bailey S. J., Winyard P. G., Blackwell J. R. (2011). Influence of N-acetylcysteine administration on pulmonary O_2_ uptake kinetics and exercise tolerance in humans. *Respiratory Physiology & Neurobiology*.

[52] Ferreira L. F., Campbell K. S., Reid M. B. (2011). N-acetylcysteine in handgrip exercise: plasma thiols and adverse reactions. *International Journal of Sport Nutrition and Exercise Metabolism*.

[53] Bridgeman M. M. E., Marsden M., MacNee W., Flenley D. C., Ryle A. P. (1991). Cysteine and glutathione concentrations in plasma and bronchoalveolar lavage fluid after treatment with N-acetylcysteine. *Thorax*.

[54] Arranz L., Fernández C., Rodríguez A., Ribera J. M., De la Fuente M. (2008). The glutathione precursor N-acetylcysteine improves immune function in postmenopausal women. *Free Radical Biology and Medicine*.

[55] Sen C. K., Rankinen T., Vaisanen S., Rauramaa R. (1994). Oxidative stress after human exercise: effect of N-acetylcysteine supplementation. *Journal of Applied Physiology*.

[56] Sen C. K., Atalay M., Hanninen O. (1994). Exercise-induced oxidative stress: glutathione supplementation and deficiency. *Journal of Applied Physiology*.

[57] Lee R., West D., Phillips S. M., Britz-McKibbin P. (2010). Differential metabolomics for quantitative assessment of oxidative stress with strenuous exercise and nutritional intervention: thiol-specific regulation of cellular metabolism with N-acetyl-L-cysteine pretreatment. *Analytical Chemistry*.

[58] Macneil L. G., Baker S. K., Stevic I., Tarnopolsky M. A. (2011). 17*β*-estradiol attenuates exercise-induced neutrophil infiltration in men. *American Journal of Physiology-Regulatory Integrative and Comparative Physiology*.

[59] Sandström M. E., Zhang S.-J., Bruton J. (2006). Role of reactive oxygen species in contraction-mediated glucose transport in mouse skeletal muscle. *The Journal of Physiology*.

[60] Sun Y., Qi Z., He Q. (2015). The effect of treadmill training and N-acetyl-L-cysteine intervention on biogenesis of cytochrome c oxidase (COX). *Free Radical Biology and Medicine*.

[61] Kyriakides C., Austen W., Wang Y. (1999). Skeletal muscle reperfusion injury is mediated by neutrophils and the complement membrane attack complex. *American Journal of Physiology-Cell Physiology*.

[62] Atalay M., Seene T., Hänninen O., Sen C. K. (1996). Skeletal muscle and heart antioxidant defences in response to sprint training. *Acta Physiologica Scandinavica*.

[63] Cobley J. N., McGlory C., Morton J. P., Close G. L. (2011). N-acetylcysteine's attenuation of fatigue after repeated bouts of intermittent exercise: practical implications for tournament situations. *International Journal of Sport Nutrition and Exercise Metabolism*.

[64] McKenna M. J., Medved I., Goodman C. A. (2006). N-acetylcysteine attenuates the decline in muscle Na+, K+-pump activity and delays fatigue during prolonged exercise in humans. *The Journal of Physiology*.

[65] Gomes-Marcondes M. C. C., Tisdale M. J. (2002). Induction of protein catabolism and the ubiquitin-proteasome pathway by mild oxidative stress. *Cancer Letters*.

[66] Agten A., Maes K., Smuder A., Powers S. K., Decramer M., Gayan-Ramirez G. (2011). N-Acetylcysteine protects the rat diaphragm from the decreased contractility associated with controlled mechanical ventilation. *Critical Care Medicine*.

[67] Servais S., Letexier D., Favier R., Duchamp C., Desplanches D. (2007). Prevention of unloading-induced atrophy by vitamin E supplementation: links between oxidative stress and soleus muscle proteolysis?. *Free Radical Biology and Medicine*.

[68] Weiss S. J. (1989). Tissue destruction by neutrophils. *The New England Journal of Medicine*.

[69] Gores-Lindholm A. R., LeBlanc M. M., Causey R. (2013). Relationships between intrauterine infusion of N-acetylcysteine, equine endometrial pathology, neutrophil function, post-breeding therapy, and reproductive performance. *Theriogenology*.

[70] Lasram M. M., Lamine A. J., Dhouib I. B. (2014). Antioxidant and anti-inflammatory effects of N-acetylcysteine against malathion-induced liver damages and immunotoxicity in rats. *Life Sciences*.

[71] Vasu V. T., De Cruz S. J., Houghton J. S. (2011). Evaluation of thiol-based antioxidant therapeutics in cystic fibrosis sputum: focus on myeloperoxidase. *Free Radical Research*.

[72] Rochat T., Lacroix J.-S., Jornot L. (2004). N-acetylcysteine inhibits Na+ absorption across human nasal epithelial cells. *Journal of Cellular Physiology*.

[73] Murakami S., Kurihara S., Titchenal C. A., Ohtani M. (2010). Suppression of exercise-induced neutrophilia and lymphopenia in athletes by cystine/theanine intake: a randomized, double-blind, placebo-controlled trial. *Journal of the International Society of Sports Nutrition*.

[74] Geudens N., Van De Wauwer C., Neyrinck A. P. (2007). N-acetyl cysteine pre-treatment attenuates inflammatory changes in the warm ischemic murine lung. *The Journal of Heart and Lung Transplantation*.

[75] Barnes P. J., Shapiro S. D., Pauwels R. A. (2003). Chronic obstructive pulmonary disease: molecular and cellular mechanisms. *The European Respiratory Journal*.

[76] Whyte M. K. B., Hardwick S. J., Meagher L. C., Savill J. S., Haslett C. (1993). Transient elevations of cytosolic free calcium retard subsequent apoptosis in neutrophils in vitro. *The Journal of Clinical Investigation*.

[77] Csontos C., Rezman B., Foldi V. (2011). Effect of N-acetylcysteine treatment on the expression of leukocyte surface markers after burn injury. *Burns*.

[78] Radomska-Leśniewska D. M., Sadowska A. M., Van Overveld F. J., Demkow U., Zieliński J., De Backer W. A. (2006). Influence of N-acetylcysteine on ICAM-1 expression and IL-8 release from endothelial and epithelial cells. *Journal of Physiology and Pharmacology*.

[79] Brandolini L., Bertini R., Bizzarri C. (1996). IL-1*β* primes IL-8-activated human neutrophils for elastase release, phospholipase D activity, and calcium flux. *Journal of Leukocyte Biology*.

[80] Woo C.-H., Yoo M.-H., You H.-J. (2003). Transepithelial migration of neutrophils in response to leukotriene B4 is mediated by a reactive oxygen species-extracellular signal-regulated kinase-linked cascade. *Journal of Immunology*.

[81] Abe T., Yamamura K., Gotoh S., Kashimura M., Higashi K. (1998). Concentration-dependent differential effects of *N*-acetyl-l-cysteine on the expression of HSP70 and metallothionein genes induced by cadmium in human amniotic cells. *Biochimica et Biophysica Acta (BBA)—General Subjects*.

[82] Dobashi K., Aihara M., Araki T. (2001). Regulation of LPS induced IL-12 production by IFN-*γ* and IL-4 through intracellular glutathione status in human alveolar macrophages. *Clinical and Experimental Immunology*.

[83] Víctor V. M., Rocha M., De la Fuente M. (2003). Regulation of macrophage function by the antioxidant N-acetylcysteine in mouse-oxidative stress by endotoxin. *International Immunopharmacology*.

[84] Lu Y., Qin W., Shen T. (2011). The antioxidant N-acetylcysteine promotes atherosclerotic plaque stabilization through suppression of rage, MMPs and NF-*κ*B in ApoE-deficient Mice. *Journal of Atherosclerosis and Thrombosis*.

[85] Wang Y., Zeigler M. M., Lam G. K. (2007). The role of the NADPH oxidase complex, p38 MARK, and Akt in regulating human monocyte/macrophage survival. *American Journal of Respiratory Cell and Molecular Biology*.

[86] Yi K., Chung T. Y., Hyon J. Y., Koh J. W., Wee W. R., Shin Y. J. (2011). Combined treatment with antioxidants and immunosuppressants on cytokine release by human peripheral blood mononuclear cells—chemically injured keratocyte reaction. *Molecular Vision*.

[87] Oddera S., Silvestri M., Sacco O., Eftimiadi C., Rossi G. A. (1994). N-Acetylcysteine enhances in vitro the intracellular killing of *Staphylococcus aureus* by human alveolar macrophages and blood polymorphonuclear leukocytes and partially protects phagocytes from self-killing. *The Journal of Laboratory and Clinical Medicine*.

[88] Quadrilatero J., Hoffman-Goetz L. (2004). N-Acetyl-L-cysteine prevents exercise-induced intestinal lymphocyte apoptosis by maintaining intracellular glutathione levels and reducing mitochondrial membrane depolarization. *Biochemical and Biophysical Research Communications*.

[89] Hoffman-Goetz L., Quadrilatero J. (2003). Treadmill exercise in mice increases intestinal lymphocyte loss via apoptosis. *Acta Physiologica Scandinavica*.

[90] Nielsen H. B., Secher N. H., Kappel M., Pedersen B. K. (1998). N-acetylcysteine does not affect the lymphocyte proliferation and natural killer cell activity responses to exercise. *The American Journal of Physiology*.

[91] Kucuk O., Stoner-Picking J., Yachnin S. (1992). Inhibition of NK cell-mediated cytotoxicity by oxysterols. *Cellular Immunology*.

[92] Pedersen B. K., Kharazmi A., Svenson M. (1988). Down-regulation of natural killer cell activity by autologous polymorphonuclear leucocytes. *Allergy*.

[93] Krüger K., Frost S., Most E., Völker K., Pallauf J., Mooren F. C. (2009). Exercise affects tissue lymphocyte apoptosis via redox-sensitive and Fas-dependent signaling pathways. *American Journal of Physiology—Regulatory Integrative and Comparative Physiology*.

[94] Tsai K., Hsu T.-G., Hsu K.-M. (2001). Oxidative DNA damage in human peripheral leukocytes induced by massive aerobic exercise. *Free Radical Biology and Medicine*.

[95] Steensberg A., Morrow J., Toft A. D., Bruunsgaard H., Pedersen B. K. (2002). Prolonged exercise, lymphocyte apoptosis and F2-isoprostanes. *European Journal of Applied Physiology*.

[96] Lin Y.-S., Kuo H.-L., Kuo C.-F., Wang S.-T., Yang B.-C., Chen H.-I. (1999). Antioxidant administration inhibits exercise-induced thymocyte apoptosis in rats. *Medicine and Science in Sports and Exercise*.

[97] Hartmann A., Nieß A. M., Grünert-Fuchs M., Poch B., Speit G. (1995). Vitamin E prevents exercise-induced DNA damage. *Mutation Research Letters*.

[98] Lagranha C. J., Senna S. M., de Lima T. M. (2004). Beneficial effect of glutamine on exerciseinduced apoptosis of rat neutrophils. *Medicine and Science in Sports and Exercise*.

[99] Chang W.-K., Yang K. D., Chuang H., Jan J.-T., Shaio M.-F. (2002). Glutamine protects activated human T cells from apoptosis by up-regulating glutathione and Bcl-2 levels. *Clinical Immunology*.

[100] Will Y., Kaetzel R. S., Brown M. K., Fraley T. S., Reed D. J. (2002). In Vivo reversal of glutathione deficiency and susceptibility to in Vivo dexamethasone-induced apoptosis by N-acetylcysteine and L-2-oxothiazolidine-4-carboxylic acid, but not ascorbic acid, in thymocytes from *γ*-glutamyltranspeptidase-deficient knockout mice. *Archives of Biochemistry and Biophysics*.

[101] Chiba T., Takahashi S., Sato N., Ishii S., Kikuchi K. (1996). Fas-mediated apoptosis is modulated by intracellular glutathione in human T cells. *European Journal of Immunology*.

[102] Tonomura N., McLaughlin K., Grimm L., Goldsby R. A., Osborne B. A. (2003). Glucocorticoid-induced apoptosis of thymocytes: requirement of proteasome-dependent mitochondrial activity. *Journal of Immunology*.

[103] Mooren F. C., Blöming D., Lechtermann A., Lerch M. M., Völker K. (2002). Lymphocyte apoptosis after exhaustive and moderate exercise. *Journal of Applied Physiology*.

[104] Baeuerle P. A., Henkel T. (1994). Function and activation of NF-*κ*B in the immune system. *Annual Review of Immunology*.

[105] Reddy Avula C. P., Muthukumar A. R., Zaman K., McCarter R., Fernandes G. (2001). Inhibitory effects of voluntary wheel exercise on apoptosis in splenic lymphocyte subsets of C57BL/6 mice. *Journal of Applied Physiology*.

[106] Jain S. K., Velusamy T., Croad J. L., Rains J. L., Bull R. (2009). l-Cysteine supplementation lowers blood glucose, glycated hemoglobin, CRP, MCP-1, and oxidative stress and inhibits NF-*κ*B activation in the livers of Zucker diabetic rats. *Free Radical Biology and Medicine*.

[107] Hayakawa M., Miyashita H., Sakamoto I. (2003). Evidence that reactive oxygen species do not mediate NF-*κ*B activation. *EMBO Journal*.

[108] Pajonk F., Riess K., Sommer A., McBride W. H. (2002). N-acetyl-L-cysteine inhibits 26S proteasome function: Implications for effects on NF-*κ*B activation. *Free Radical Biology and Medicine*.

[109] Qiu Y., Zhang J., Liu Y. (2013). The combination effects of acetaminophen and N-acetylcysteine on cytokines production and NF-*κ*B activation of lipopolysaccharide-challenged piglet mononuclear phagocytes in vitro and in vivo. *Veterinary Immunology and Immunopathology*.

[110] Shi J., Sun X., Lin Y. (2014). Endothelial cell injury and dysfunction induced by silver nanoparticles through oxidative stress via IKK/NF-*κ*B pathways. *Biomaterials*.

[111] Ji L. L., Gomez-Cabrera M.-C., Steinhafel N., Vina J. (2004). Acute exercise activates nuclear factor (NF)-*κ*B signaling pathway in rat skeletal muscle. *The FASEB Journal*.

[112] Kramer H. F., Goodyear L. J. (2007). Exercise, MAPK, and NF-*κ*B signaling in skeletal muscle. *Journal of Applied Physiology*.

[113] Nieman D. C., Henson D. A., Butterworth D. E. (1997). Vitamin C supplementation does not alter the immune response to 2.5 hours of running. *International Journal of Sport Nutrition*.

